# Preclinical evaluation of a synthetic peptide vaccine against SARS-CoV-2 inducing multiepitopic and cross-reactive humoral neutralizing and cellular CD4 and CD8 responses

**DOI:** 10.1080/22221751.2021.1978823

**Published:** 2021-09-27

**Authors:** Belén Aparicio, Noelia Casares, Josune Egea, Marta Ruiz, Diana Llopiz, Sheila Maestro, Cristina Olagüe, Gloria González-Aseguinolaza, Cristian Smerdou, Ascensión López-Díaz de Cerio, Susana Inogés, Felipe Prósper, José R. Yuste, Francisco Carmona-Torre, Gabriel Reina, Juan J. Lasarte, Pablo Sarobe

**Affiliations:** a Centro de Investigación Médica Aplicada (CIMA), Universidad de Navarra, Pamplona, Spain; b Centro de Investigación Biomédica en Red de Enfermedades Hepáticas y Digestivas (CIBEREHD), Pamplona, Spain; c IdiSNA, Instituto de Investigación Sanitaria de Navarra, Pamplona, Spain; d Clínica Universidad de Navarra, Pamplona, Spain; e Centro de Investigación Biomédica en Red de Cáncer (CIBERONC), Pamplona, Spain

**Keywords:** SARS-CoV-2, peptide vaccine, B-cell epitopes, neutralizing antibodies, T-cell epitopes

## Abstract

Identification of relevant epitopes is crucial for the development of subunit peptide vaccines inducing neutralizing and cellular immunity against SARS-CoV-2. Our aim was the characterization of epitopes in the receptor-binding domain (RBD) of SARS-CoV-2 spike (S) protein to generate a peptide vaccine. Epitope mapping using a panel of 10 amino acid overlapped 15-mer peptides covering region 401-515 from RBD did not identify linear epitopes when tested with sera from infected individuals or from RBD-immunized mice. However, immunization of mice with these 15-mer peptides identified four peptides located at region 446-480 that induced antibodies recognizing the peptides and RBD/S1 proteins. Immunization with peptide 446-480 from S protein formulated with Freund’s adjuvant or with CpG oligodeoxinucleotide/Alum induced polyepitopic antibody responses in BALB/c and C56BL/6J mice, recognizing RBD (titres of 3 × 10^4^–3 × 10^5^, depending on the adjuvant) and displaying neutralizing capacity (80–95% inhibition capacity; *p *<* *0.05) against SARS-CoV-2. Murine CD4 and CD8T-cell epitopes were identified in region 446-480 and vaccination experiments using HLA transgenic mice suggested the presence of multiple human T-cell epitopes. Antibodies induced by peptide 446-480 showed broad recognition of S proteins and S-derived peptides belonging to SARS-CoV-2 variants of concern. Importantly, vaccination with peptide 446-480 or with a cyclic version of peptide 446-488 containing a disulphide bridge between cysteines 480 and 488, protected humanized K18-hACE2 mice from a lethal dose of SARS-CoV-2 (62.5 and 75% of protection; *p *<* *0.01 and *p *<* *0.001, respectively). This region could be the basis for a peptide vaccine or other vaccine platforms against Covid-19.

## Introduction

In December 2019, an outbreak of patients with pneumonia-like symptoms was reported in China [1]. It was caused by a highly transmissible and pathogenic coronavirus denominated Severe acute respiratory syndrome coronavirus 2 (SARS-CoV-2) [2], which has been responsible for a subsequent pandemic of acute respiratory disease, named “coronavirus disease 2019” (COVID-19). Although two highly pathogenic coronavirus, severe acute respiratory syndrome coronavirus (SARS-CoV) and Middle East respiratory syndrome coronavirus (MERS-CoV), emerged in 2002 and 2012 [3], SARS-CoV-2 has exceeded them in terms of the number of infected people and areas affected. This rapid widespread has resulted in a global pandemic and public health crisis, affecting also the global economy [4]. This has led to the development of vaccines based on different platforms, and some of them have been approved [5–8] and are being rolled out in many countries. Approved vaccines include those based on mRNA [5,6] and recombinant adenoviral vectors [7,8], while others, such as those based on proteins [9], are being tested in phase III clinical trials and could be potentially approved in the near future. Based on the previous experience obtained with SARS-CoV in animal models [10] and humans [11], the spike (S) protein has been the antigen of choice for most vaccines. S protein contains S1 and S2 subunits, implicated in the interaction with the cellular receptor angiotensin-converting enzyme-2 (ACE-2) and in the fusion with cell membrane for viral entry [12]. The importance of S protein in virus/cell interaction, its immunogenicity at the B and T-cell level [13,14], including its capacity to induce neutralizing antibodies in infected patients [15], have been important factors to consider this protein as the main target in vaccine design. Accordingly, S protein-based vaccines include features aimed at maintaining its structure and stability, and mimic thus the antigenicity found in viral particles [16,17]. Among the regions of S protein, the receptor-binding domain (RBD) located at the S1 subunit is of paramount importance, since it is involved in virus/cell interactions [18,19] and antibodies against this region have neutralizing capacity [20]. Thus, some vaccines have focused on this domain, containing different antigen versions [21].

Besides the relevance of neutralizing antibodies, T-cell responses are also of paramount importance, since CD4T-cells are necessary for T helper (Th) dependent antibody responses, where they promote the generation of memory B-cells, isotype switching and affinity maturation, features highly important in prophylactic vaccines [22]. Moreover, CD4T-cells collaborate to promote CD8T-cell responses with capacity to kill virus-infected cells.

Antigens included in protein vaccines or encoded by nucleic acid- or viral vector-based vaccines are complex, but B and T lymphocytes usually recognize small epitope regions of these antigens, which can be mimicked by peptides. Therefore, several peptide-based vaccines containing B and/or T-cell epitopes have been designed for other diseases [23]. Although some B-cell epitopes contained in antigenic sites of proteins have a conformational nature, there are other epitopes present in linear regions, providing thus the opportunity for vaccine design based on the use of linear B-cell epitopes [24]. Importantly, T-cell epitopes always consist of short peptides presented by MHC molecules [25].

Synthetic peptide vaccines have been pursued for years [26]. In addition to the immunological issues mentioned above. Some practical features, such as their ease and flexibility of design, chemical synthesis and manufacturing, and their high stability have been considered as advantages for these immunogens [27]. On the other side, the potential inability of short peptides fragments to mimic conformational B-cell epitopes contained in antigenic sites of folded proteins, and their lack of signals activating innate immunity, requiring thus their combination with immunostimulatory adjuvants [28], have become relevant obstacles in the development of peptide vaccines. Therefore, our goal was to design and test a peptide-based vaccine, to be directly used against SARS-CoV-2 or as a component in other vaccination platforms. To this aim, we have first carried out B cell epitope mapping experiments, by using a 15-mer peptide panel from RBD with sera from Covid-19 patients and from mice immunized with RBD, as well as by immunizing mice with this peptide panel, leading to the identification of the polyepitopic region 446-480 with capacity to induce neutralizing and cross-reactive antibodies. Also, we have tested the presence of murine T cell epitopes by immunizing with peptide 446-480, as well as the presence of potential human T cell epitopes in S1 protein by using human leukocyte antigens (HLA) binding prediction algorithms and by immunizing HLA transgenic mice expressing representative class I and class II alleles HLA-A*02.01 and HLA-DRB1*01. These experiments have led to the design of a polyepitopic peptide in RBD sequence 446-480 with capacity to induce cross-reactive neutralizing antibodies and T-cell responses.

## Materials and methods

### Peptides and proteins

Peptide libraries comprising 21 overlapping 15-mer peptides (with 10 amino acids overlap) belonging to 401-515 region of RBD and 48 20-mer peptides (with 10 amino acids overlap; amino acids 1-490) from S1 protein (original Wuhan isolate [2]) were synthesized in an automatic APEX 396 multiple peptide synthesizer (Aapptec LLC, Louisville, KY). Peptide purity was analysed by HPLC. S1 peptides 446-480, 446-488cc (containing a disulfide bridge between cysteines 480 and 488) and Th peptides FIS (FISEAIIHVLHSR) and pan HLA DR-binding epitope PADRE (AKFVAAWTLKAAA), with a purity >90%, were purchased from Genecust (Boynes, France). S1 (ref Z03501-100) and RBD (ref Z03483-100) proteins expressed in human cells, as well as variant RBD proteins with E484 K (ref Z03535-100), N501Y (ref Z03533-100) or L452R (ref Z03603) mutations were purchased from Genscript (Leiden, The Netherlands).

### Human sera

The protocols and informed consent forms were approved (24/04/2020) by the research and ethics committee of the Clínica Universidad de Navarra (Pamplona, Spain) (ref # 2020-067 and 2020-090) prior to study implementation. Serum samples were obtained from outpatients not requiring hospitalization (*n* = 5) and from hospitalized (*n* = 12) or convalescent (*n* = 8) patients with active or previous SARS-CoV-2 infection confirmed by RT-PCR (Supplementary Table S1).

### Mice

BALB/c and C57BL/6J mice (8 weeks old) were obtained from Envigo (Barcelona, Spain). HHD-DR1 mice (B2^mtm1Unc^ H2-Ab1^tm1Doi^ Tg(HLA-A2/H2-D/B2M)1Bpe Tg(HLA-DR1)/Orl, transgenic for human HLA-A*02.01 and HLA-DRB1*01 molecules) were obtained from Dr F. Lemmonier (Institute Pasteur, Paris, France) and bred in our facilities. K18-hACE2 mice (B6.Cg-Tg(K18-ACE2)2Prlmn/J) expressing human ACE-2 (8 weeks old) were purchased from The Jackson Laboratory (Bar Harbor, ME). Mice were maintained in pathogen-free conditions according to the guidelines for animal care of our Institutional Review Board (protocol 025-20).

### Immunization experiments

The following immunization protocols were used: a) Antibody induction capacity of each peptide from a panel covering 401-515 region of RBD was initially tested in BALB/c mice (*n* = 3/peptide), immunized according to our protocol previously described [29]. Briefly, they were injected intraperitoneally with the 15-mer peptide to be tested (50 μg/mouse, equivalent to 27 nmoles) plus the Th peptide FIS (50 μg/mouse) [29] and complete Freund’s adjuvant (Difco Laboratories; Detroit, MI). An additional group received as antigen 1 μg of RBD protein. They were boosted at days 30 and 45 with the same dose of antigen in incomplete Freund’s adjuvant. b) In experiments carried out using as immunogen peptide 446-480 from S1 protein, BALB/c or C57BL/6J mice (*n* = 5/group) were immunized with 110 μg (27 nmoles) of 446-480 peptide with or without the Th peptide FIS (for BALB/c mice) or PADRE peptide (for C57BL/6J mice). When using adjuvants approved for human use, mice received by intramuscular route different doses (27–110 μg) of peptide 446-480 or 446-488cc in combination with 10 μg of the CpG-containing oligodeoxinucleotide ODN1018 (Sigma; Darmstadt, Germany) and 50 μg of alum (Invivogen; Toulouse, France) and boosted at day 21, as previously described for murine studies [30]. c) For T-cell activation experiments with peptide 446-480, BALB/c and C57BL/6J mice were immunized with 110 μg of peptide combined with Freund’s adjuvants or with CpG ODN1018/alum as above. d) Screening of T cell immunogenicity of 20-mer peptides covering region 1-490 was carried out in HHD-DR1 mice, injected subcutaneously with pools of seven-eight peptides (100 μg each) in combination with poly(I:C) (50 μg) (GE Healthcare; Chicago, IL) and agonistic anti-CD40 antibodies (50 μg) (Bioxcell; Lebanon, NH). In this last case, they were boosted one week later and sacrificed seven days after the last immunization as described [31].

### ELISA

Antibodies were detected by ELISA by using as antigens synthetic peptides and proteins as described [29]. Plates were coated with peptides (1 µg/well) or proteins (0.1 µg/well) and different serum dilutions were analysed. A similar protocol was applied to human sera, but using a 1/5000 solution of biotinylated goat anti-human IgG (Sigma; Darmstadt, Germany) as a secondary antibody. Murine serum endpoint titres were calculated individually for each mouse as the last dilution with an optical density above that obtained with control sera plus 2 standard deviations.

### ELISPOT

An ELISPOT assay (BD-Biosciences; San Diego, CA) measuring Interferon (IFN)-γ secreting T-cells was used to determine the generation of T-cell responses induced by vaccines. Briefly, spleens were obtained from each immunized animal, individually homogenized and erythrocytes were lysed with red blood cell (RBC) lysing buffer. Splenocytes (8 × 10^5^/well) were stimulated with peptides (10 μg/ml) for 24 h in capture antibody-coated plates. In some cases, stimulation experiments were carried out in the presence of anti-CD4 or anti-CD8 antibodies (100 μg/ml). After extensive washing, plates were incubated with detection antibody for 2 h, subsequently washed and spots developed by using 3-Amino-9-ethylcarbazole substrate. Spot-forming cells were counted automatically using an ImmunoSpot automated counter (CTL-Immunospot; Bonn, Germany).

### Flow cytometry

Splenocytes from immunized mice were stimulated *in vitro* with peptides (10 μg/ml) in the presence of GolgiStop and GolgiPlug (BD-Biosciences) and 4 h later they were harvested and stained with antibodies CD3ϵ-Percp-Cy5 (145-2C11), CD4-FITC (RM4-5), CD8-BV421 (53-6.7) from BioLegend (San Diego, CA). Next, cells were fixed and permeabilized using BD Cytofix/Cytoperm™ Fixation/Permeabilization Kit for intracellular staining with IFNγ-PE (XMG1.2). Dead cells were excluded from the analysis using Maleimide (PromoCell; Heidelberg, Germany). Samples were acquired with a Cytoflex (Beckman Coulter; Indianapolis, IN) flow cytometer. All data were analysed using FlowJo software (FlowJo LLC; Ashland, OR).

### Prediction with HLA binding algorithms

The sequence comprising 1-490 from SARS-CoV-2 S1 protein was uploaded in NetMHCpan 4.1 (http://www.cbs.dtu.dk/services/NetMHCpan/) and NetMHCIIpan 4.0 (http://www.cbs.dtu.dk/services/NetMHCpan/) to identify binder peptides. For HLA class I binding, peptides with a length of 8 to 11 amino acids were analysed, selecting those with a % Rank > 2. For HLA class II binding, 15-mer peptides with a % Rank > 10 were selected.

### Neutralization assays

SARS-CoV-2 virus (isolate NAVARRA-2473) [32] used for these assays was obtained in April 2020 from the nasal sample of a COVID-19 patient hospitalized in Clínica Universidad de Navarra, after obtaining the patient´s informed consent and Regional Government permits. We first grew the virus in confluent Vero-E6 cells (ATCC® CRL-1586™) and supernatants were collected at 72 h post-inoculation, when a clear cytopathic effect was evident. The virus was then titrated using a lysis plate assay using Vero-E6 cell monolayers, resulting in a titre of 4.3 × 10^7^ plaque-forming units (pfu)/ml. For neutralization assays, Vero-E6 cells grown in 96-well plates with Eagle's Minimum Essential Medium plus 10% foetal bovine serum and antibiotics were used. Before infection, serum dilutions were incubated for 1 h at 37 °C with SARS-CoV-2 virus at a multiplicity of infection of 0.05. Then this mix was added to confluent monolayers of Vero-E6 cells and incubated for 1 h at 37°C. After removing the inoculum, medium containing the same serum dilutions was added to infected Vero-E6 cells. One day later supernatants were collected and SARS-CoV-2 was quantified using the Real-time Fluorescent RT-qPCR Kit for Detecting 2019-nCoV (BGI, Copenhagen, Denmark).

### In vivo protection experiments

K18-hACE2 transgenic mice (*n* = 7–8/group) were immunized with peptide 446-480 or with the cyclic peptide 446-488cc (27 nmoles/mouse) and CpG ODN1018/alum as above. Mice receiving RBD (1 μg/mouse) plus CpG ODN1018/alum adjuvants or only adjuvants were used as controls. They were boosted at day 21 and at day 38 were challenged with 10^5^ pfu of SARS-CoV-2 (isolate NAVARRA-2473). Virus was administered under anesthesia by intranasal route (10 μl/nostril). Body weight and mortality was monitored daily and mice were euthanized when reaching a 25% weight loss.

### Statistical analyses

Groups were analysed using Student’s *t*-test and one-way ANOVA with Bonferroni’s multiple comparison test. Survival was analysed with the Log-rank test. *p *<* *0.05 was taken to represent statistical significance.

## Results

### Recognition of linear epitopes in the RBD of S1 protein

To identify linear epitopes in RBD we first used serum samples from 19 infected individuals and 10 controls and analysed their reactivity against a panel of 21 15-mer overlapping peptides encompassing amino acids 401-515 of the RBD. Although all infected individuals had antibodies recognizing RBD and none of the controls displayed reactivity against this domain (*p *<* *0.0001), there was a great heterogeneity in terms of responses against peptides (Supplementary Figure S1A). Nineteen out of 21 peptides (90.4%; 95% confidence interval 98.3–71.1%) were recognized by sera from infected patients, whereas 9 out of 21 peptides (42.8%; 95% confidence interval 63.5–24.5%) were targeted by antibodies from control sera. However, we could not identify any immunodominant peptide recognized by most patients, with none of the peptides being recognized by >20% of sera. These results suggest that there is no clear reactivity pattern in sera of infected individuals specifically recognizing linear B-cell epitopes in this RBD region.

To obtain more information of antibodies against linear epitopes in RBD, we next immunized mice with RBD. Despite a strong reactivity against RBD in these sera, only residual responses were observed, directed against region 411-435 (covered by three peptides) and region 461-480 (covered by two peptides) (Supplementary Figure S1B). Thus, both in the natural infection and in immunized animals most anti-RBD antibodies are not directed against linear B-cell epitopes located in this region.

### Immunization with peptides from RBD induces anti-peptide antibodies that recognize the viral protein

**Figure 1.**
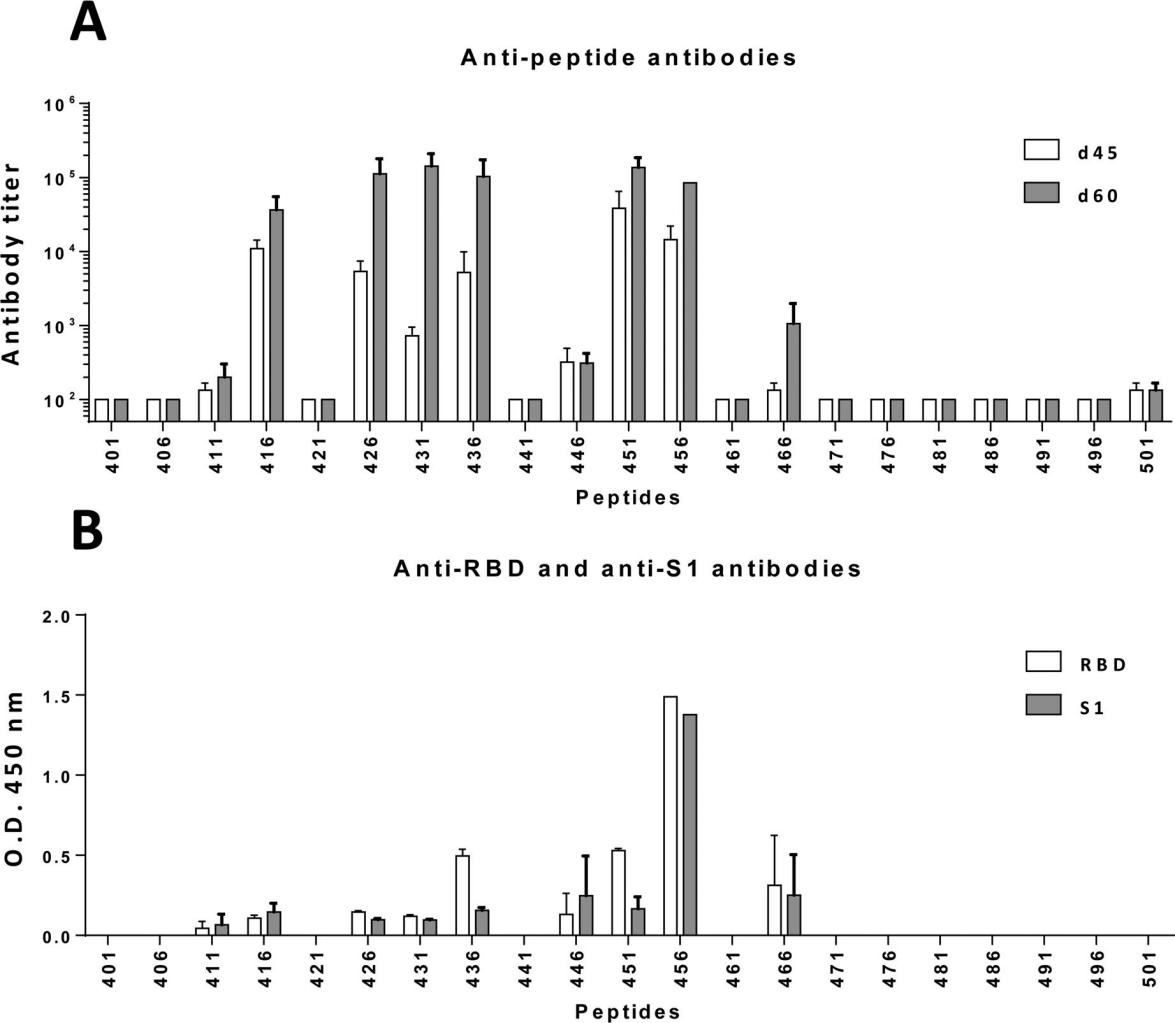
***Immunogenicity of short synthetic peptides from RBD and recognition of viral proteins by anti-peptide antibodies.*** BALB/c mice (*n *=* *3/group) were immunized with individual 15-mer peptides plus the T helper epitope FIS. (A) Sera were obtained at days 45 and 60 and tested against the immunizing peptide. (B) Recognition of RBD and S1 proteins by sera from each group obtained at day 60 was also analysed by ELISA. Results correspond to the mean + SEM values of individual mice.

The lack of antibodies induced against linear B-cell epitopes prompted us to use a strategy based on epitope mapping by immunizing with short synthetic peptides, as we have demonstrated for other proteins [29]. We immunized mice with each of the 21 15-mer peptides plus the Th epitope FIS (presented by BALB/c MHC class II molecules) and anti-peptide antibodies were analysed. Antibodies were induced against 9 out of 21 peptides (42%) grouped in regions 411-430, 426-450 and 446-480 (Figure 1(A)). Interestingly, despite the lack of recognition of peptides by sera induced by viral infection in humans or after immunization with RBD in mice, some peptides induced antibodies that recognized not only the peptide, but also RBD and S1 proteins (Figure 1(B)). Although equivalent anti-peptide titres were observed for regions 411-430, 426-450 and 446-480, the best recognition of the proteins was centred in region 446-480, suggesting that it could be considered for further immunogen design.

### Peptide 446-480 from RBD induces multiepitopic responses against RBD

After identifying the capacity of several peptides belonging to region 446-480 to induce antibodies cross-recognizing viral proteins, we designed a longer peptide encompassing residues 446-480. Since a 35-mer peptide may adopt a conformation more similar to that present in the original protein than that displayed by the shorter 15-mer peptides, we tested whether peptide 446-480 was better recognized by sera from patients. However, it was not recognized by sera from 13 patients (*p* = 0.1, patients vs healthy controls) who had strong anti-RBD antibodies (*p *<* *0.001, patients vs healthy controls) (Supplementary Figure S2A). Recognition of peptide 446-480 by sera from mice immunized with the 15-mer peptides contained in the 35-mer was poor but measurable. However, sera from mice immunized with RBD recognized much better peptide 446-480 (mean titre 8200) (Supplementary Figure S2B) than 15-mer peptides (mean titres below 200, as shown in Supplementary Figure 1B, suggesting that peptide 446-480 may better mimic the structure found in the original protein than shorter peptides.

**Figure 2.**
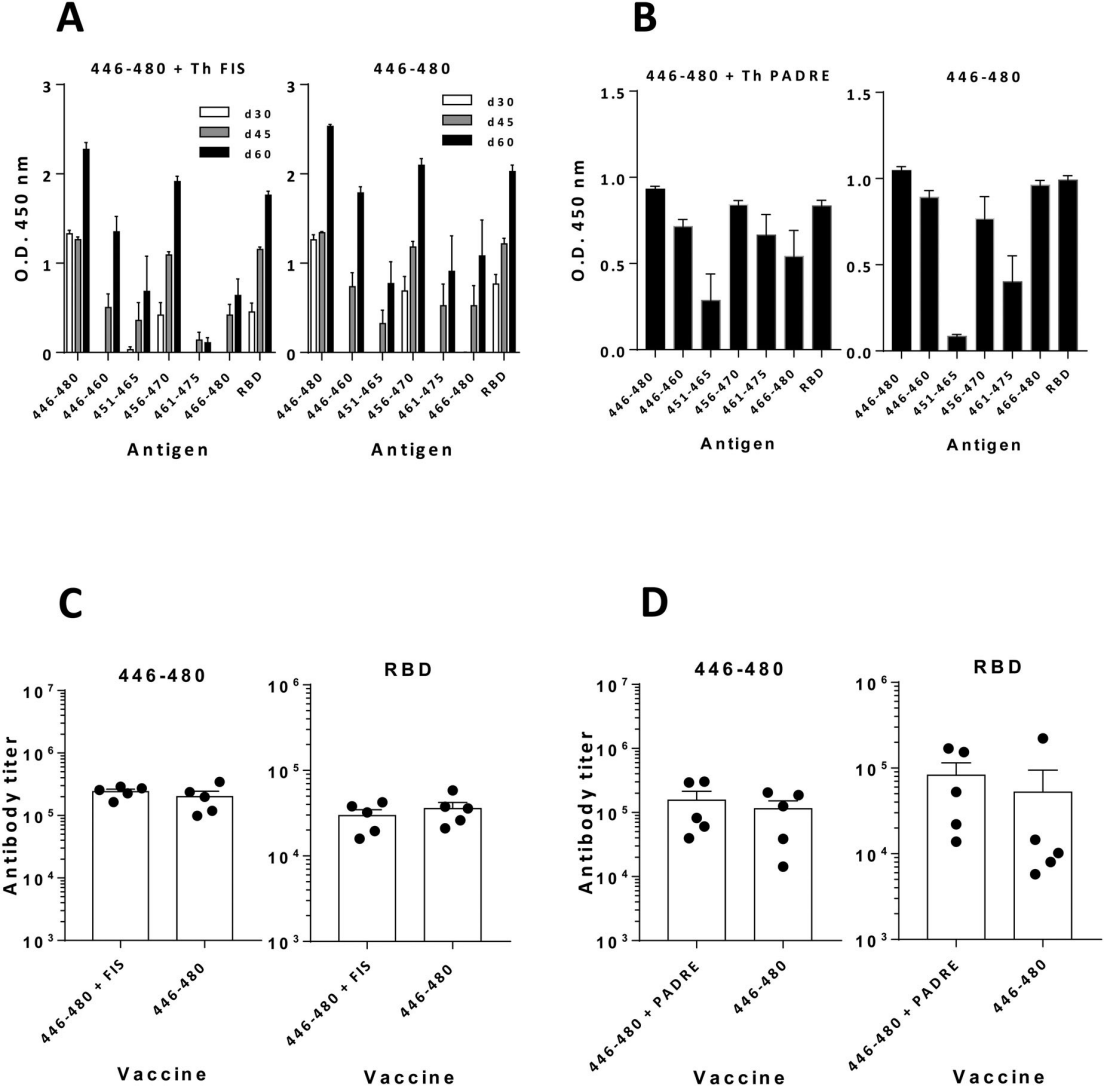
***Peptide 446-480 induces multiepitopic antibody responses that recognize SARS-CoV-2 RBD.*** (A) BALB/c mice (*n *=* *5) were immunized with peptide 446-480 with or without the Th epitope FIS. Responses against 446-480, individual 15-mer peptides and RBD were analysed by ELISA. (B) C57BL/6J mice (*n *=* *5) were immunized as in A but with the Th epitope PADRE and tested at day 60 against the same panel of antigens. Sera from BALB/c (C) and C57BL/6J (D) mice were titrated against peptide 446-480 and against RBD. Results correspond to the mean + SEM values of individual mice.

We next immunized mice with peptide 446-480 in the presence or absence of the Th epitope FIS. Strong antibody responses against the whole 446-480 sequence, the five 15-mer peptides (focused mainly on peptide 456-470) and RBD were observed (Figure 2(A)). Interestingly, immunization in the absence of the Th epitope FIS induced equivalent responses. Similar experiments in C57BL/6J mice using the Th peptide PADRE [33] also showed strong responses against peptide 446-480, RBD, and the 15-mer peptides contained in 446-480 (Figure 2(B)). Again, 446-480 was also immunogenic in the absence of the exogenously added Th peptide. These results suggest the presence of Th epitopes within 446-480 peptide. Titration experiments demonstrated that immunization with peptide 446-480 induced antibodies with titres above 10^5^ for 446-480 peptide and 3-5 × 10^4^ for RBD in BALB/c (Figure 2(C)) and C57BL/6J mice (Figure 2(D)). No benefit was provided by co-administration of Th peptides, both when testing responses against peptide 446-480 (*p* = 0.52 in BALB/c; *p* = 0.42 in C57BL/6J mice) or against RBD (*p* = 0.22 in BALB/c; *p* = 0.69 in C57BL/6J mice).

### Antibodies induced by peptide 446-480 neutralize SARS-CoV-2 in vitro

**Figure 3.**
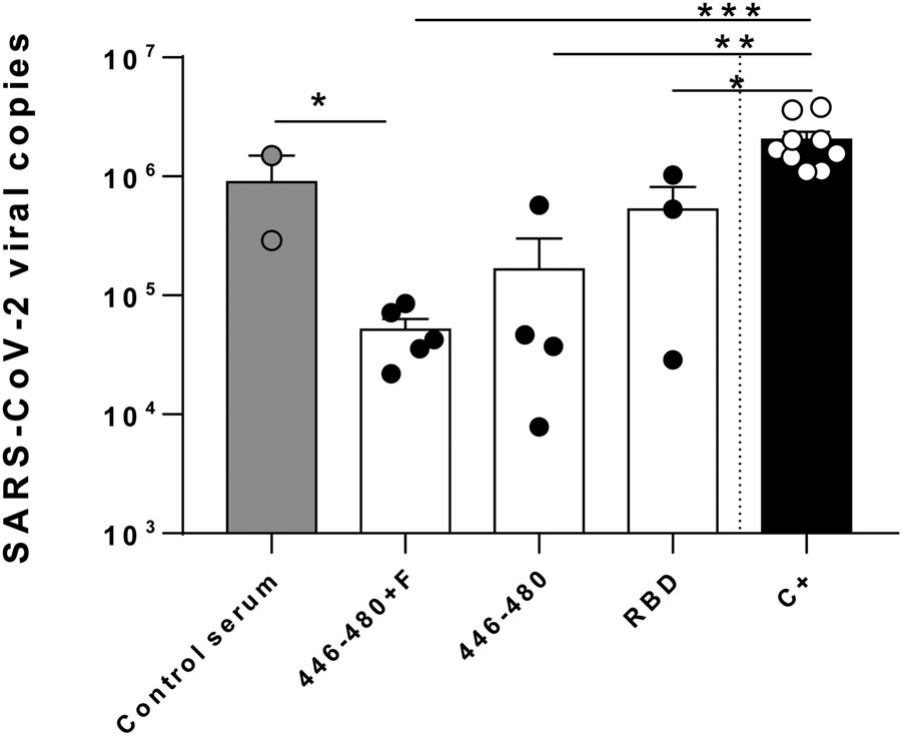
***Antibodies induced by immunization with peptide 446-480 neutralize SARS-CoV-2 in vitro.*** SARS-CoV-2 viral particles were incubated with media (C+), control serum or with sera from mice immunized with peptide 446-480, 446-480 + Th FIS or with RBD (*n *=* *3–5 sera/group) and used to infect Vero-E6 cells. One day later viral load was determined by PCR. (*p <* *0.05; **p <* *0.01; ***p <* *0.001).

To test the neutralizing capacity of sera obtained from mice immunized with peptide 446-480 we incubated Vero-E6 cells with SARS-CoV-2 particles in the presence of these sera. Sera from mice immunized with 446-480 peptide with or without the Th epitope significantly reduced viral infectivity by 80 to 95% at a 1/200 dilution, as opposed to sera from non-immunized mice (negative control sera) (*p *<* *0.05) (Figure 3). We also included some sera from mice immunized with RBD, which had an intermediate neutralization capacity.

### Immunization with peptide 446-480 in adjuvants approved for human use

**Figure 4.**
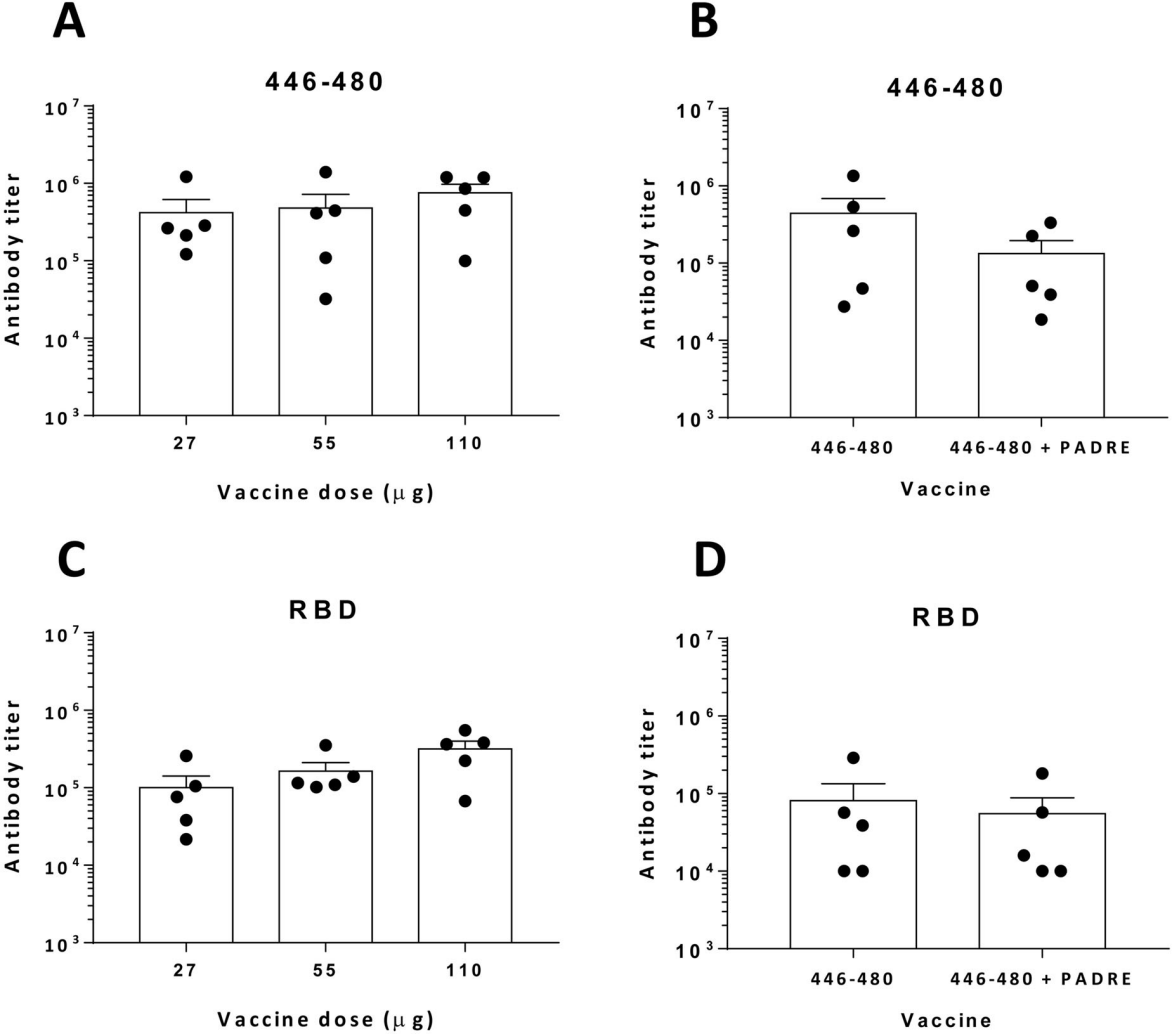
***Immunization with peptide 446-480 and CpG ODN1018/Alum adjuvants induces potent humoral responses that recognize RBD.*** Peptide 446-480 in combination with adjuvants CpG ODN1018/Alum was used to immunize BALB/c mice (*n *=* *5/group) at different doses (A, C) or C57Bl/6J mice (*n *=* *5/group) with or without the Th peptide PADRE (B, D). Sera were obtained at day 35 and tested against peptide 446-480 or against RBD. Results correspond to the mean + SEM values of individual mice.

After identifying the target region 446-480, we tested its immunogenicity using adjuvants approved for human use. We combined this peptide with the TLR9 ligand CpG ODN1018 and alum, to promote antibody induction and T-cell responses [30]. Since we already knew the capacity of peptide 446-480 to induce antibodies in BALB/c mice in the absence of exogenous Th peptides, this combination was omitted. Moreover, we also immunized C57BL/6J mice with or without the Th peptide PADRE. In BALB/c mice, strong antibody responses were observed at the three doses tested (Figure 4(A)), without any statistically significant difference between doses (*p* = 0.54; 27 vs 110 μg doses). For C57BL/6J mice, similar antibody titres were induced with or without the presence of the Th peptide (*p* = 0.56) (Figure 4(B)). In all cases, antibodies induced by the peptide could also recognize the RBD with titres around 10^5^ (Figure 4(C,D)) without differences between groups (*p* = 0.06 in BALB/c mice; *p* = 0.22 in C57BL/6J mice). We also tested adjuvants Addavax, a squalene-based emulsion similar to MF59®, and Quil A, a saponin adjuvant used in human studies. Although some responses were induced when using these compounds, they were not as efficient as the CpG/alum combination (Supplementary Figure S3).

### Peptide 446-480 induces antibodies cross-recognizing variants of concern

**Figure 5.**
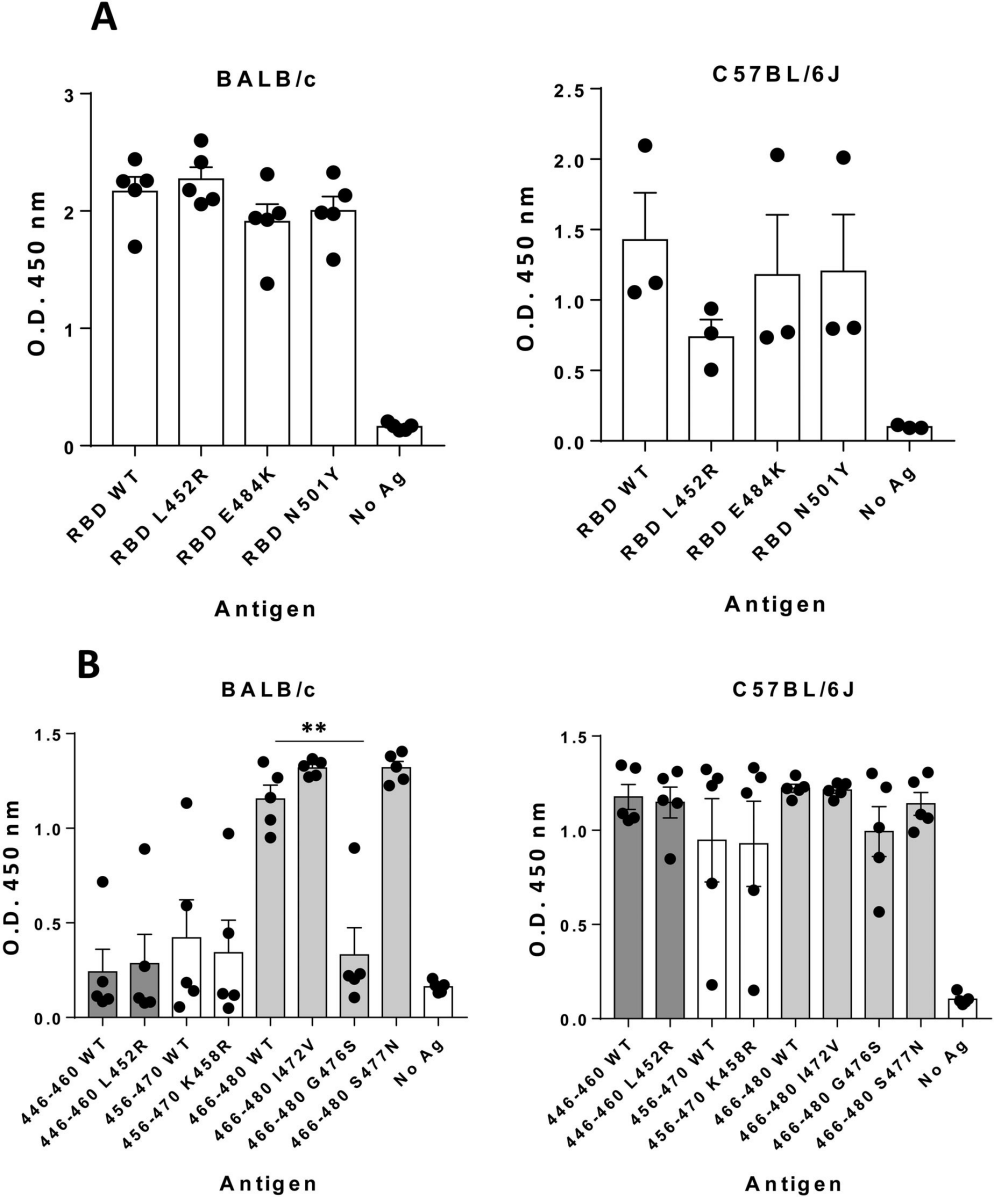
***Antibodies induced by peptide 446-480 cross-recognize antigens from SARS-CoV-2 variants of concern.*** Sera (*n *=* *5/group; 1/5000 dilution) obtained from BALB/c mice (left panels) or C57BL/6J mice (right panels) at day 35 after immunization with peptide 446-480 in CpG ODN1018/Alum were tested against recombinant RBD proteins with mutations L452R, E484 K or N501Y (A) or against synthetic peptides with mutations contained within the 446-480 region (B) (**p <* *0.01). Results correspond to the mean + SEM values of individual mice.

Recent reports have shown similar or decreased recognition and neutralization of new viral variants with mutations at RBD by sera from previously infected or vaccinated individuals [34]. When testing the capacity of sera from BALB/c and C57BL/6J mice immunized with peptide 446-480 to recognize recombinant RBD proteins bearing the common mutations L452R, E484 K or N501Y, we observed similar recognition to that of WT sequence, without statistically significant differences (Figure 5(A)). Moreover, we checked recognition of less common variants located inside the 446-480 region. By using synthetic peptides as antigens (Supplementary Table S2), we found that, although poorer recognition was observed for mutation G476S in BALB/c mice (*p *<* *0.01 vs WT), antibodies against 446-480 displayed strong cross-recognition of peptides with mutations L452R, K458R, I472 V and S477N (Figure 5(B)).

### Peptide 446-480 contains T-cell epitopes

**Figure 6.**
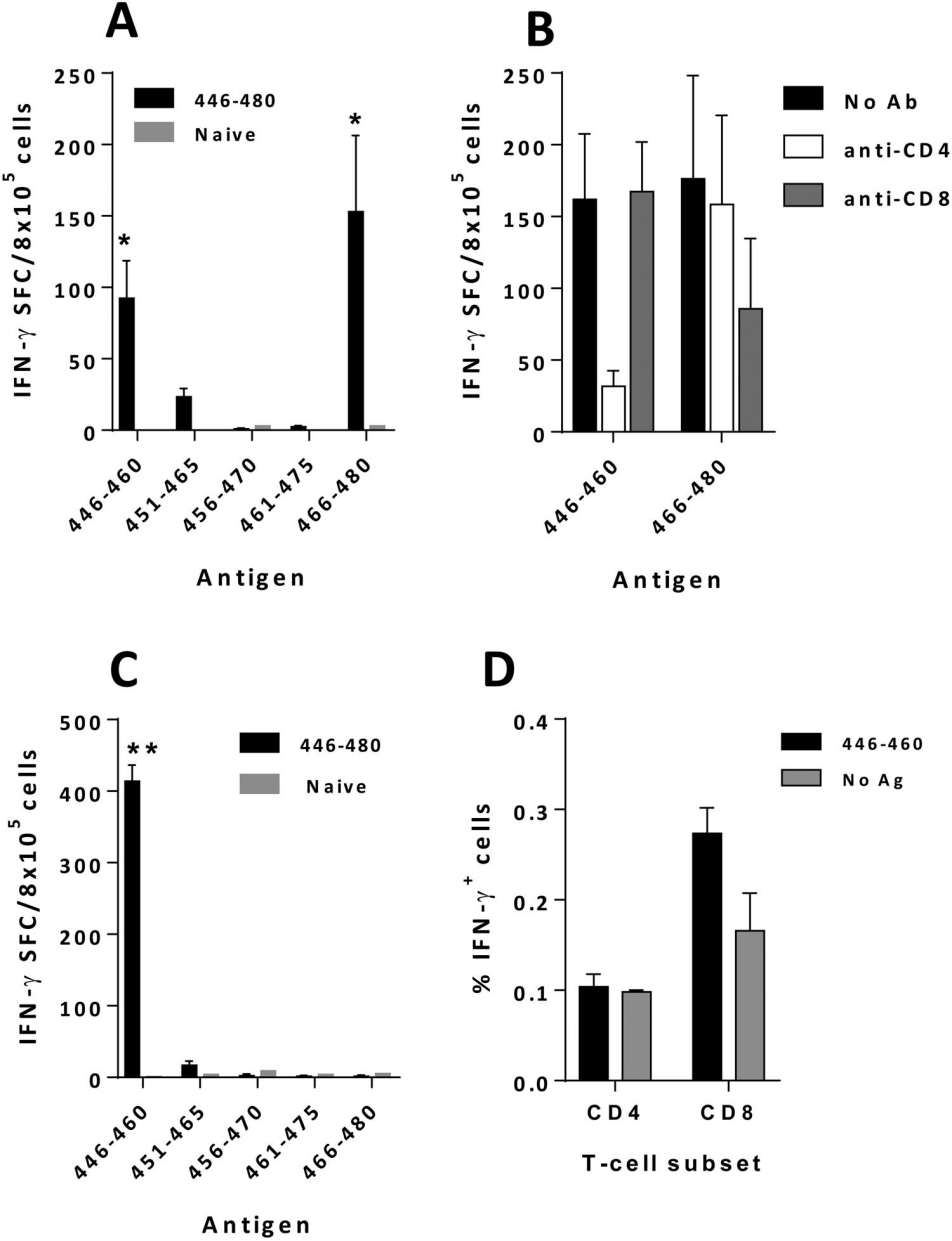
***Peptide 446-480 contains T-cell epitopes in different murine strains.*** BALB/c (A) and C57BL/6J (C) mice (*n *=* *5/group) were immunized with peptide 446-480 with CpG ODN1018/Alum adjuvants and 2 weeks after the last immunization splenocytes were stimulated with 15-mer peptides contained within 446-480. T-cell responses were evaluated by an IFN-gamma ELISPOT. (B) Splenocytes from mice used in A were stimulated with peptides 446-460 or 466-480 in the presence of blocking anti-CD4 or anti-CD8 antibodies and IFN-gamma production was measured. (D) Cells from mice used in C were stimulated with peptide 446-460 and the proportion of CD4 and CD8 T-cells producing IFN-gamma was determined by flow cytometry. (*p <* *0.05; **p <* *0.01). Results correspond to the mean + SEM values of individual mice.

Induction of antibodies in both mouse strains in the absence of exogenous Th peptides suggested the presence of T-cell epitopes within 446-480, in agreement with predictions obtained with MHC binding algorithms (Supplementary Table S3). To identify these T-cell epitopes, splenocytes from BALB/c mice immunized with 446-480 in CpG/Alum were stimulated with 15-mer peptides. Peptides 446-460 and 466-480 were identified as T-cell epitopes (Figure 6(A)). Equivalent results were obtained when using Freund’ adjuvants (Supplementary Figure S4A). Blocking experiments with antibodies against CD4 or CD8 showed that 446-460 was clearly recognized by CD4T-cells, whereas CD8T-cells seemed to recognize peptide 466-480 (Figure 6(B)). Similar analyses of T-cell responses in C57BL/6J mice immunized with 446-480 in CpG/Alum also demonstrated a strong response against peptide 446-460 (Figure 6(C)), in this case, mediated by CD8T-cells (Figure 6(D)). Combination of peptide 446-480 with the T helper peptide PADRE induced similar T cell responses, targeting peptide 446-460 (Supplementary Figure S4B).

### T-cell epitopes in S1 subunit

**Figure 7.**
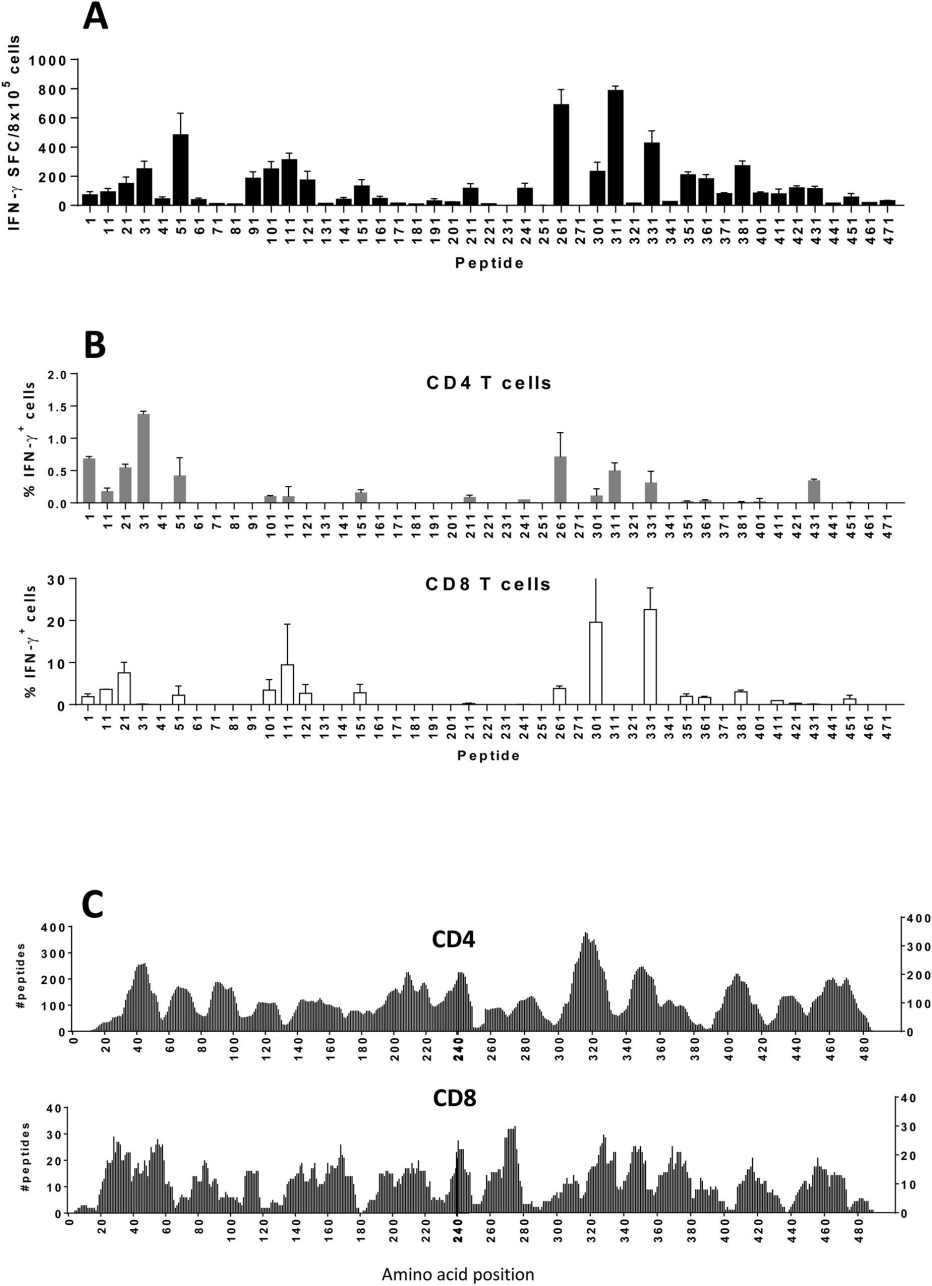
***Peptide 446-480 is located in a region containing potential T-cell epitopes present by human MHC molecules.*** (A) HHD-DR1 mice (*n *=* *4/group) were immunized with 20-mer peptides covering sequence 1-490 from S1. Splenocytes were stimulated with individual peptides and responses were determined by ELISPOT. (B) Stimulated cells were also analysed by flow cytometry to identify CD4 and CD8T-cells responsible for IFN-gamma production. Results correspond to the mean + SEM values of individual mice. (C) The number of peptides from region 1-490 in S1, potentially presented by a panel of MHC class I and class II molecules, was calculated by using the NetMHCpan and NetMHCIIpan algorithms.

Besides T-cell epitopes contained in 446-480, we were interested in other T-cell epitopes located in the 1-490 region of S1, potentially useful as additional components of a subunit peptide vaccine. As a humanized *in vivo* model of immunogenicity we used HHD-DR1 mice, transgenic for human HLA-A*02.01 and HLA-DRB1*01 molecules. Mice were immunized with pools of 20-mer peptides and splenocytes stimulated in vitro with the peptides. IFN-gamma production showed that almost half of the peptides were immunogenic (Figure 7(A)), with weak responses directed against peptides located at the 446-480 region. Flow cytometry experiments demonstrated that immunogenic epitopes induced CD4 or CD8 responses, and in some cases, like for peptides 21, 261 and 331, both cell subsets were activated (Figure 7(B)).

Since HLA-A2.01 and HLA-DRB101 are alleles that are expressed only by a proportion of individuals, we predicted *in silico* those regions containing peptides with potential capacity to bind to a set of 12 and 54 HLA class I and class II molecules (Supplementary Table S4), representative of a wider proportion of the population. This yielded several “hot” regions with peptides potentially able to be presented by a high number of HLA alleles. Of note, some immunogenic CD4 and CD8 peptides identified in HHD mice coincided with regions containing peptides presentable by a higher number of HLA alleles (Figure 7(C)). Finally, although HLA-A*02.01- and HLA-DRB1*01-restricted responses against peptides contained in the 446-480 region observed in HHD mice were weak, this region was included in a prominent peak, suggesting that, as shown above for murine MHC molecules, it presumably contains T-cell epitopes presented by a wide number of HLA class I and class II molecules.

### Peptide 446-480 and the cyclic peptide 446-488cc protect K18-hACE2 transgenic mice from a lethal dose of SARS-CoV-2

Once demonstrated the immunogenic capacity of peptide 446-480 to induce neutralizing antibodies we evaluated the efficacy of this peptide vaccine candidate in K18-hACE2 mice challenged with a lethal dose of SARS-CoV-2. Based on the crystal structure of RBD region complexed with the ACE2 receptor S protein [35], and motivated by the presence of two cysteines at positions 480 and 488 that may participate in binding to the host receptor, we also evaluated in this murine model the immunogenicity and neutralization capacity of a vaccine based on the cyclic peptide 446-488cc (containing a disulphide bridge between these two cysteines). Previous vaccination experiments in BALB/c and C57BL/6J mice using 446-488cc peptide and CpG ODN1018/adjuvants demonstrated its immunogenicity at the B and T cell level (Supplementary Figure S5A,B). Moreover, antibodies induced in BALB/c or C57BL/6J mice by 446-488cc recognized RBD protein with mutation E484 K similarly to WT RBD (Supplementary Figure S5C).

**Figure 8.**
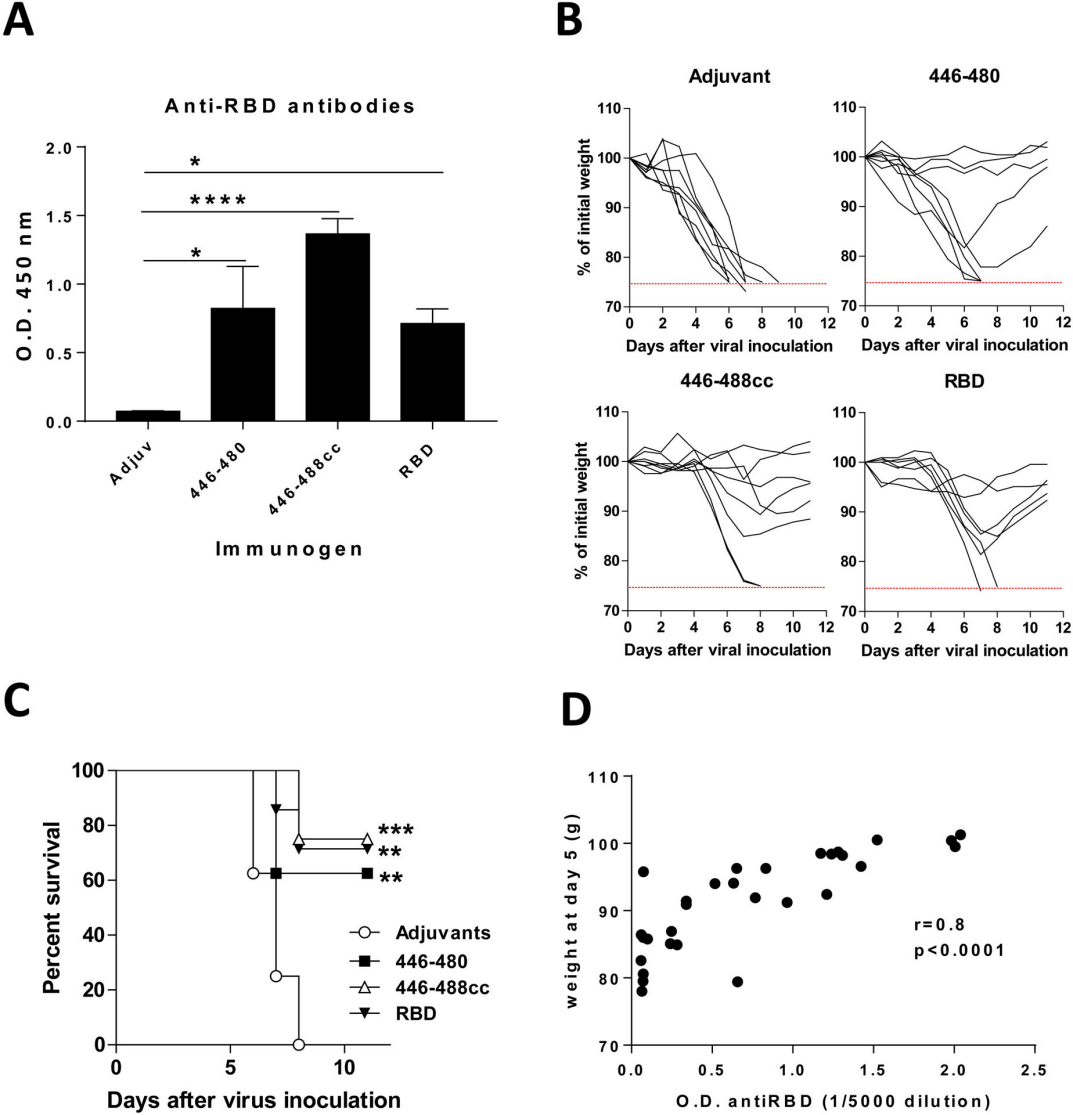
***Peptide 446-480 and the cyclic peptide 446-488cc protect K18-hACE2 transgenic mice from a lethal dose of SARS-CoV-2.*** K18-hACE2 mice (*n *=* *7–8/group) were immunized with peptide 446-480, 446-488cc, RBD protein plus CpG ODN1018/Alum adjuvants or adjuvants alone. Anti-RBD antibodies (1/5000 dilution) were determined at day 35 by ELISA (A). Mice were inoculated with 10^5^ pfu/mouse of SARS-CoV-2 and body weight (B) and survival (C) was monitored. Correlation between antibody levels and body weight at day 5 after viral inoculation was analysed (D). (*p <* *0.05; **p <* *0.01; ***p <* *0.001; ****p <* *0.0001).

Thus, K18-hACE2 mice were vaccinated twice with 27 nmoles of peptide 446-480 or 446-488cc in CpG/Alum. As controls, we included mice immunized with RDB protein plus adjuvants or mice with adjuvants in the absence of any antigen. Sera obtained from K18-hACE2 mice vaccinated with the different immunogens contained antibodies that recognized RBD, with the highest levels observed in the group treated with 446-488cc (Figure 8(A)). Ten days after the second immunization, mice were challenged with 1 × 10^5^ pfu/mouse of SARS-CoV-2 (isolate NAVARRA-2473) by the intranasal route. Body weight and mortality was monitored for 11 days postchallenge. All mice immunized with adjuvants alone showed a dramatic loss of body weight (more than 25%) and died within 8 days postchallenge. However, 62.5% of mice vaccinated with 446-480 (*p* = 0.0073), 75% of mice immunized with 446-488cc peptide (*p* = 0.0004) and 71% of mice immunized with RBD (*p* = 0.0026) recovered after a limited initial loss of weight and survived (Figure 8(B,C)). Interestingly, the protective effect observed in these groups was related to the antibody levels induced in these mice, since pre-inoculation anti-RBD antibodies had a strong correlation with protection against weight loss (Figure 8(D)). Thus, peptide vaccination was efficacious to control SARS-CoV-2 morbidity and lethality in this mouse model of infection.

## Discussion

Identification of relevant epitopes is the basis for the development of peptide vaccines. Common vaccines induce polyepitopic responses with anti-pathogen properties, but they may also induce non-neutralizing “enhancing” antibodies which facilitate infection, as occurs in several viral infections including SARS-CoV [36] or even recently shown in SARS-CoV-2 [37]. Therefore, to generate safe and efficacious vaccines, characterization of epitopes inducing neutralizing antibodies and proper cellular immunity is needed. Thus, we focused on RBD, due to the capacity of RBD protein vaccines to induce strong neutralizing antibody titres [21]. The poor identification of linear B-cell epitopes using 15-mer peptides [38], as opposed to results obtained with longer peptides [24,39], forced us to adopt an alternative mapping strategy. In this way we found that half of the tested peptides were immunogenic, and that some of them induced antibodies that recognized the original protein, validating this approach [29]. The potential flexible conformations adopted by 15-mer immunogens may explain the capacity of these peptide-induced antibodies to recognize the peptide and the protein, as opposed to protein-induced antibodies, which may be induced by a more rigid structure, allowing less conformational possibilities.

Immunogenic epitopes inducing anti-RBD antibodies were mainly located in the 446-480 region, and this 35-mer peptide was better recognized by sera from RBD-immunized mice than the 15-mers, suggesting that it could be potentially folded more similarly to RBD. Thus, when combined with different adjuvants, this peptide-induced potent multiepitopic antibody responses that recognized recombinant RBD and had a clear neutralizing capacity, in agreement with the involvement that residues located in this region have on the interaction with ACE-2 receptor [40] and with already characterized neutralizing monoclonal antibodies [41].

Immunogenicity of peptide 446-480 in the absence of exogenous Th peptides suggested the presence of intrinsic T-cell epitopes. They were identified as recognized by CD4 and CD8T-cells, indicating that it could simultaneously activate humoral and cellular responses, making unnecessary the presence of exogenous antigens. In addition to neutralizing antibodies against SARS-CoV-2, cellular immunity may have important implications, since it has been shown a good correlation with humoral immunity [42], although these responses may vary depending on the clinical setting [14,43], with uncoordinated responses in some cases [44]. Regarding CD8T-cells, usually involved in the clearance of virus-infected cells, they have been detected in patients, targeting different proteins, including spike [45,46]. Some CD8T-cells induced by the infection have a “stem cell memory” and naïve precursor phenotype, suggesting that they have limited clonal expansion and/or differentiation, and indicating that proper vaccination may overcome this situation [47]. Regarding the 446-480 region, despite the lack of T-cell epitopes observed with HHD-DR1 mice, prediction analyses carried out by us and others [48,49] suggest the presence of several HLA class I and class II epitopes in this peptide, recently confirmed for CD4 epitopes as an important immunodominant region [50]. Therefore, identification of a region within the RBD encompassing B-cell epitopes with capacity to induce neutralizing antibodies and CD4 and CD8T-cell epitopes provides a great opportunity to design a single peptide vaccine.

Emergence of new viral strains of concern is posing a new problem for the protection of exposed and vaccinated individuals [51–53], and for efficacy of antibody-based therapies [54]. Poorer cross-recognition has been reported, with varying degrees depending on the strain analysed. Considering the need of vaccines with the broadest protection, antibodies induced by 446-480 recognized viral proteins with mutations L452R, E484 K and N501Y, common in the Indian B.1.617, UK B.1.1.7, Brazilian P1 and P2 and South African B.1.351 variants [55,56], located out of the sequence targeted by the vaccine. Notably, they also recognized peptides with mutations occurring inside the 446-480 region, such as L452R mutation of Indian variant B.1.617, among others [57]. Our cross-recognition experiments have been done with proteins or peptides bearing single mutations, as opposed to the combination of mutations present in variants of concern, and this may be a limitation to our study due to changes in protein conformation that may modify their antigenicity. However, linear epitopes, like those identified with short peptides within 446-480 region, would be less affected than conformational epitopes, which rely on non-consecutive amino acids for their folding. This suggests that a vaccine based on peptide 446-480 could be of interest for multiple viral variants.

Vaccination against SARS-CoV-2 brings to the forefront the question of mutational escape from elicited responses. Although SARS-CoV-2 mutation rate is considered lower than in other RNA viruses [58], strong positive selection pressure exerted by vaccines may favour the appearance of escape variants [59]. Virus shedding can occur from patients with mild symptoms or asymptomatic, even in the presence of antibodies against SARS-CoV-2. Interestingly, current S antigen-based vaccines claim to induce a humoral neutralizing response with a capacity to recognize new emerging variants similar to that induced by natural infection [54]. In this way, both unvaccinated patients and vaccinated individuals are a breeding ground for the emergence of escape variants. However, if a vaccine like the one described in the present study induces a humoral immune response not elicited by natural infection, which in most cases is diverted towards immunodominant epitopes, it is tempting to speculate that the emergence of escape variants to this peptide-based vaccine will be reduced. In any case, new experiments are necessary to support this hypothesis.

In addition to its immunogenicity, safety and antiviral efficacy are important issues in vaccine development. Although we do not have experiments formally designed to test safety, no overt adverse events have been detected during the immunization experiments in the short and long term (up to 8 weeks), similarly to macroscopical pathological signs. Future experiments will be needed to ascertain safety of peptide 446-480-based vaccines.

There are currently several vaccines approved for human use that have demonstrated a good activity [5–8]. In this regard, a peptide vaccine may have additional features. Besides immunological properties demonstrated above for peptide 446-480, there are practical advantages, such as the ease and homogeneity of production, very well defined composition and stability, among others. Moreover, design and production of this type of vaccine offers a great flexibility, a favourable feature mainly considering the potential appearance of new variants with different sequences, allowing the possibility of generating polyvalent vaccines that combine sequences corresponding to the different variants [60]. However, there are some limitations that have to be considered when dealing with this type of vaccines, such as their inability to mimic conformational B cell epitopes, as we have shown when comparing recognition of 15-mer versus the 35-mer peptides, requiring in some cases the synthesis of longer sequences With this premise, and based on the crystal structure of protein S/ACE2 complex, we synthesized peptide 446-488cc, which, due to a disulphide bridge between cysteines 480 and 488, could better mimic a conformational B epitope. Indeed, immunization with this peptide resulted in higher immunogenicity and in vivo protective capacity. On the other hand, immunogenicity at the T cell level may be compromised by the absence of T cell epitopes, requiring extensive T cell epitope mapping to identify those promiscuous regions with peptides presentable by several HLA alleles. Similarly, short peptides are usually unable to activate innate immunity, requiring thus the combination with immunomodulatory adjuvants. The growing availability of these stimulatory adjuvant molecules will help future development of vaccines, where the type of response can be tailored according to the adjuvant used. Finally, our results may be useful not only for peptide vaccine design, but also for the design/improvement of new vaccines based on other platforms, such as mRNA or other vectored vaccines. Identification of regions to be included in a vaccine, targeting only relevant epitopes with capacity to induce neutralizing or cross-reactive antibodies, and not diverting the response against non-pertinent regions, may provide important clues for other vaccine platforms.

## Conclusion

In summary, we have identified region 446-488 within the RBD that induces neutralizing antibodies and cellular responses against SARS-CoV-2, with capacity to cross-recognize common escape variants and protect from viral infection, suggesting that this region could be used in a peptide vaccine against Covid-19.

## Supplementary Material

Supplementary_material_revised.docx
