# Supplementary material for: Preclinical evaluation of a synthetic peptide vaccine against SARS-CoV-2 inducing multiepitopic and cross-reactive humoral neutralizing and cellular CD4 and CD8 responses
Source: Emerg Microbes Infect. 2021 Sep 27;10(1):1931–46. doi: 10.1080/22221751.2021.1978823 (PMC8480813; doi:10.1080/22221751.2021.1978823)
Supplement: Supplementary_material_revised.docx [file TEMI_A_1978823_SM6411.docx]

**Preclinical evaluation of a synthetic peptide vaccine against SARS-CoV-2 inducing multiepitopic and cross-reactive humoral neutralizing and cellular CD4 and CD8 responses**

Belén Aparicio et al.

**Supplementary Material:**

- **Supplementary Table S1. Description of SARS-CoV-2-infected patients.**
- **Supplementary Figure S1. Recognition of 15-mer peptides from RBD by sera obtained from Covid-19 patients and from mice immunized with RBD.**
- **Supplementary Figure S2. Recognition of peptide 446-480 by sera from SARS-CoV-2 patients and from peptide immunized mice.**
- **Supplementary Figure S3. Immunization with peptide 446-480 and other adjuvants.**
- **Supplementary Table S2. List of peptide located in 446-480 region belonging to SARS-CoV-2 variants.**
- **Supplementary Table S3. List of peptides predicted as binders to MHC class I and class II molecules in C57BL/6J and BALB/c mice.**
- **Supplementary Figure S4. Induction of murine T cell responses by peptide 446-480.**
- **Supplementary Table S4. List of HLA class I and class II alleles used for prediction analyses of peptides in S1 protein.**
- **Supplementary Figure S5. Induction of humoral and cellular responses by peptide 446-488cc.**

**Supplementary Table S1. Description of SARS-CoV-2-infected patients**

| **Patient ID** | **AGE** | **SEX** | **Day Sample Collection*** | **RT-PCR**** | **Status***** |
| --- | --- | --- | --- | --- | --- |
| 1 | 62 | M | 13 | Positive | Hosp |
| 2 | 74 | M | 32 | Positive | Hosp |
| 3 | 84 | M | 32 | Positive | Hosp |
| 4 | 43 | M | 18 | Positive | Hosp |
| 5 | 55 | M | 27 | Positive | Hosp |
| 6 | 55 | M | 28 | Positive | Hosp |
| 7 | 54 | M | 26 | Positive | Hosp |
| 8 | 60 | M | 4 | Positive | Hosp |
| 9 | 20 | F | 19 | Negative | Outpatient |
| 10 | 23 | F | 25 | Negative | Outpatient |
| 11 | 52 | M | 28 | Negative | Outpatient |
| 12 | 35 | M | 37 | Positive | Outpatient |
| 13 | 67 | F | 32 | Positive | Hosp |
| 14 | 89 | M | 28 | Positive | Hosp |
| 15 | 29 | F | 14 | Negative | Outpatient |
| 16 | 47 | F | 25 | Negative | Hosp |
| 17 | 59 | F | 30 | Positive | Hosp |
| 18 | 46 | F | 16 | Positive | Conv W1 |
| 19 | 76 | F | 15 | Positive | Conv W1 |
| 20 | 65 | F | 17 | Positive | Conv W1 |
| 21 | 45 | F | 3 | Positive | Conv W1 |
| 22 | 83 | M | 34 | Positive | Conv W1 |
| 23 | 83 | M | 22 | Positive | Conv W2 |
| 24 | 57 | F | 22 | Positive | Conv W2 |
| 25 | 68 | M | 19 | Positive | Conv W2 |

(*) Day of sample collection after symptoms onset.

(**) RT-PCR status at the time of sample collection

(***) Hosp: hospitalized, Conv: convalescent after hospitalization (weeks after end of symptoms/discharge), Outpatient: individual not requiring hospitalization

**Supplementary Figure S1. Recognition of 15-mer peptides from RBD by sera obtained from Covid-19 patients and from mice immunized with RBD.**

(A) Sera from 19 patients with SARS-CoV-2 infections and 12 unexposed controls were tested against 21 15-mer peptides from RBD by ELISA. RBD was used as a positive control. Upper graph: percentage of individuals responding against each peptide in the patient and control groups; Lower graph: individual responses as a heat map. (B) BALB/c mice (n=3) were immunized with RBD protein and sera was tested by ELISA at day 60 against the panel of 21 15-mer peptides.


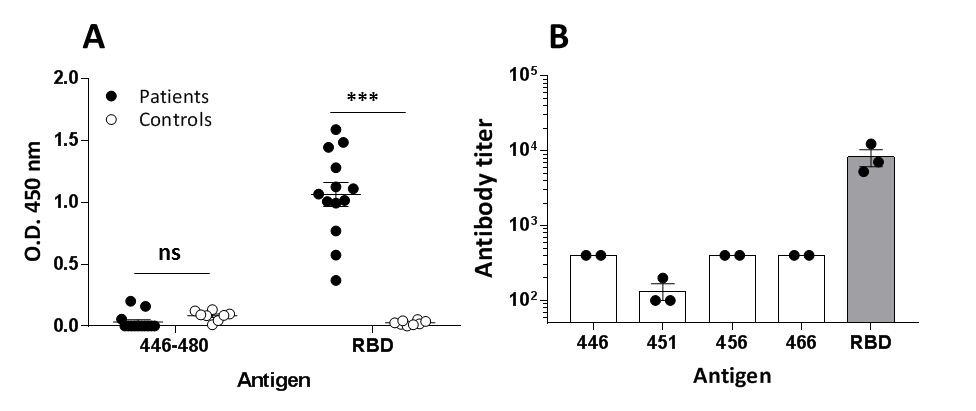


**Supplementary Figure S2. Recognition of peptide 446-480 by sera from SARS-CoV-2 patients and from peptide immunized mice.** (A) Human sera from SARS-CoV-2 patients and controls were tested against peptide 446-480 and RBD by ELISA (ns: non statistically significant; ***; P < 0.001). (B) Sera from BALB/c mice (n=3) immunized with individual 15-mer peptides contained within peptide 446-480 or immunized with RBD were tested against peptide 446-480.

**Supplementary Figure S3. Immunization with peptide 446-480 and other adjuvants.** BALB/c mice (n=4/group) were immunized with 110 μg of peptide 446-480 in combination with adjuvants Addavax (1:1 emulsion; 50 μl/mouse) and Quil A (15 μg/mouse) (Invivogen). They were boosted on days 15 and 21 and bled at different time points. Results correspond to O.D. obtained using a 1:100 serum dilution.

**Supplementary Table S2. List of peptides located in 446-480 region belonging to SARS-CoV-2 variants**

446-460 WT GGNYNYLYRLFRKSN

446-460 L452R GGNYNY**R**YRLFRKSN

456-470 WT FRKSNLKPFERDIST

456-470 K458R FR**R**SNLKPFERDIST

466-480 WT RDISTEIYQAGSTPC

466-480 I472V RDISTE**V**YQAGSTPC

466-480 G476S RDISTEIYQA**S**STPC

466-480 S477N RDISTEIYQAG**N**TPC

**Supplementary Table S3. List of peptides predicted as binders to MHC class I and class II molecules in C57BL/6J and BALB/c mice**


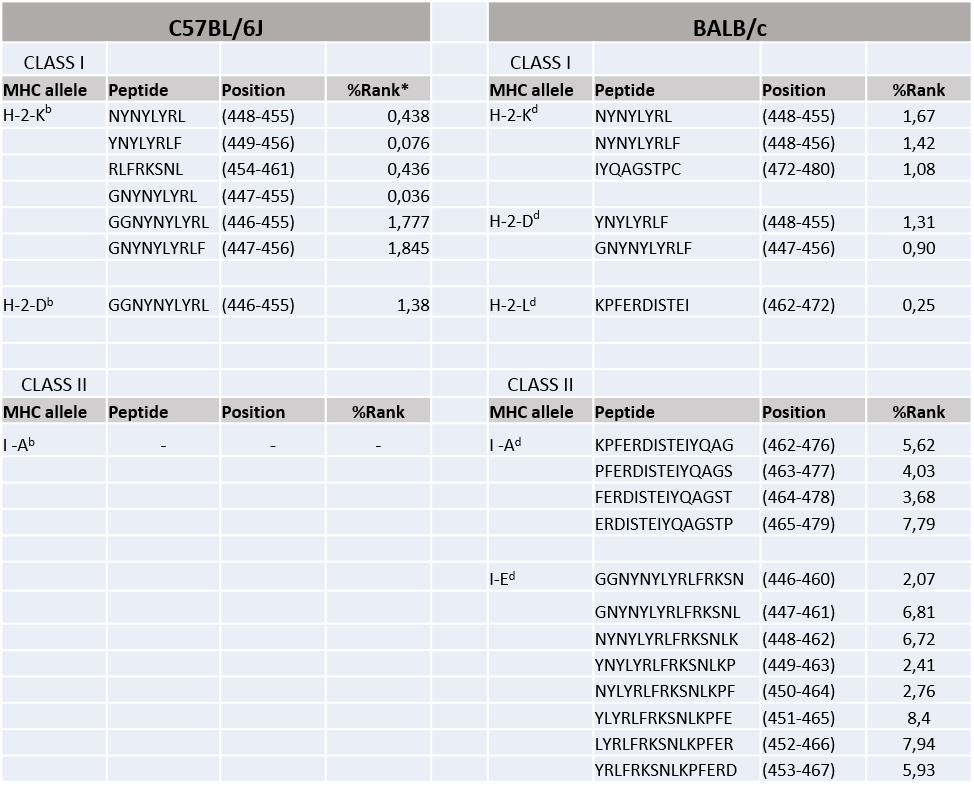


(*) Only those peptides with % Rank values <2 for class I molecules and <10 for class II molecules are shown.

**
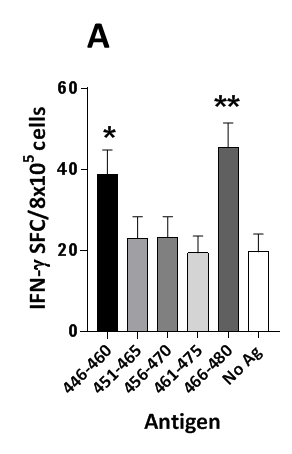

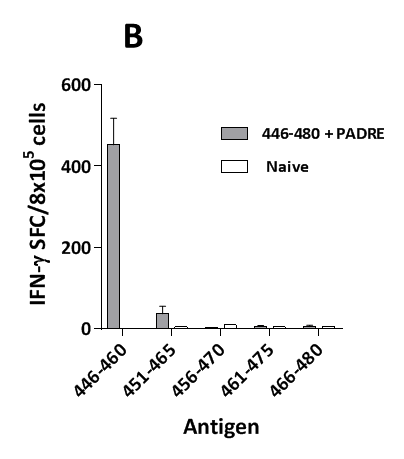
**

**Supplementary Figure S4. Induction of murine T cell responses by peptide 446-480.** (A) BALB/c mice (n=5/group) were immunized with peptide 446-480 with CFA/IFA and 2 weeks after the last immunization splenocytes were stimulated with 15-mer peptides contained within 446-480. T-cell responses were evaluated by an IFN-gamma ELISPOT.

Spleen cells from C57BL/6J immunized with peptide 446-480 with the Th peptide PADRE in CpG/Alum (n=5/group) were stimulated with 15-mer peptides and the number of IFN-gamma producing cells was measured by ELISPOT (B) whereas the proportion of CD4 and CD8 T-cells producing IFN-gamma was determined by flow cytometry (C).

**Supplementary Table S4. List of HLA class I and class II alleles used for prediction analyses of peptides in S1 protein**


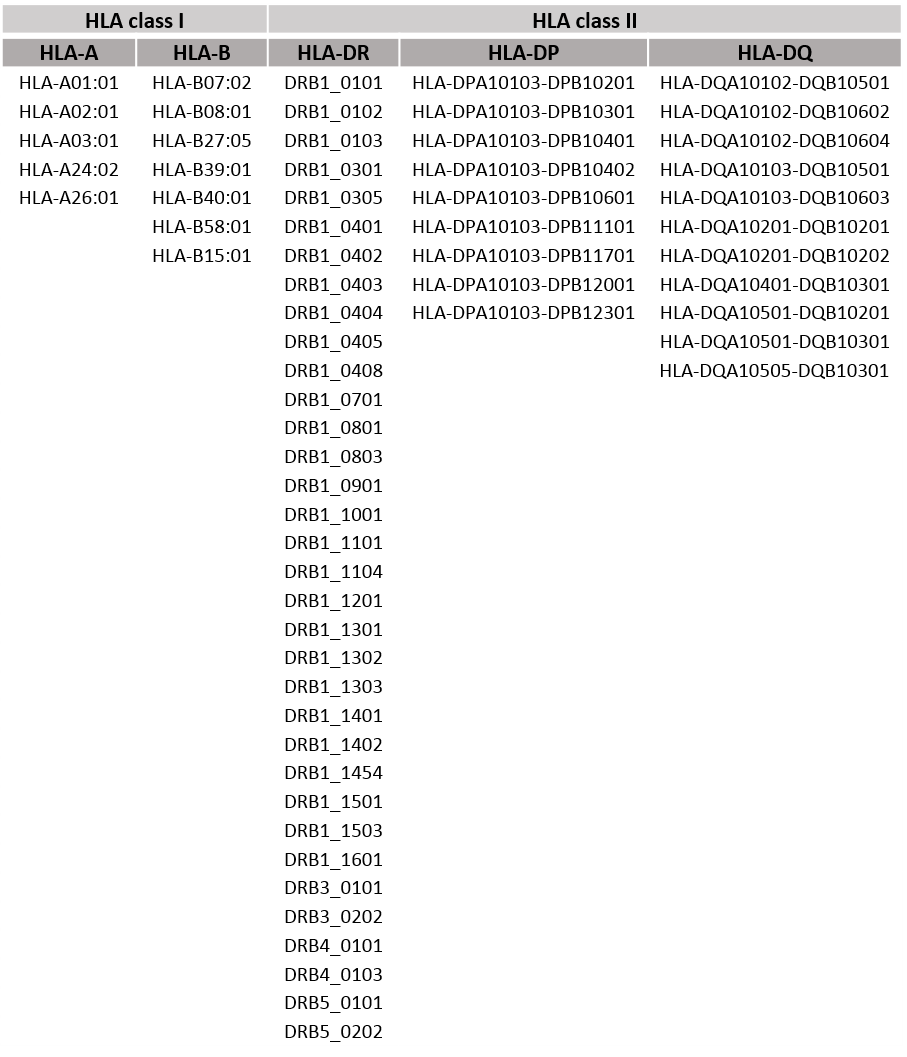


**A**

**B**

**C**

**Supplementary Figure S5. Induction of humoral and cellular responses by peptide 446-488cc.** (A) BALB/c and C57BL/6J mice (n=4-5/group) were immunized with peptide 446-488cc with CpG ODN1018/Alum adjuvants and 2 weeks after the last immunization sera was titrated against the peptide 446-488cc (left) or RBD protein (right). (B) Splenocytes from mice shown above were stimulated with peptide 446-488cc or with 15-mer peptides 446-460 and 466-480 contained within 446-480 and T-cell responses were evaluated by an IFN-gamma ELISPOT. (C) Sera shown in A (1/1000 dilution) were tested against RBD WT and RBD E484K.
